# Exploring the Impact of Prostitution on HIV/AIDS Transmission

**DOI:** 10.1155/2014/651025

**Published:** 2014-10-30

**Authors:** C. P. Bhunu, A. N. Mhlanga, S. Mushayabasa

**Affiliations:** Department of Mathematics, University of Zimbabwe, P.O. Box MP 167, Harare, Zimbabwe

## Abstract

HIV/AIDS has been somehow linked to prostitution for decades now. A mathematical model is presented to assess the link between prostitution and HIV transmission. The epidemic thresholds known as the reproduction numbers and equilibria for the model are determined and stabilities analyzed. Analysis of the reproduction numbers suggests that HIV/AIDS control using antiretroviral therapy is more effective in the absence of prostitution. Numerical simulations further show high levels of HIV/AIDS when percentage of prostitutes in the community is high. Results from this study suggest that effectively controlling HIV/AIDS requires strategies that address both prostitution and HIV/AIDS transmission. Addressing HIV/AIDS through condom use and antiretroviral therapy may not be enough to stem HIV/AIDS in the community as some drug/alcohol misusing prostitutes may not be able to negotiate for safe sex while they are in drunken stupor. Furthermore, prostitutes are likely to get infected by different HIV strains some of which may be resistant to the antiretroviral therapy regimen in use.

## 1. Introduction

Prostitution is often described as the oldest profession [1]. By definition it describes sexual intercourse in exchange for renumeration [2]. It is mainly driven by peer pressure, homelessness, drug addiction, and poverty just to mention a few of the driving forces [1]. In Zimbabwe like most African countries, prostitution although not legal, it is still common among the poverty stricken communities. A lot of Zimbabwean women are turning into prostitution in Botswana and South Africa due to poverty and unemployment in their home country [3]. Prostitution is socially stigmatized with prostitutes being more stigmatized than their male customers [4].

Prostitution is strongly associated with drug/alcohol misuse [5–9]. Some individuals turn into prostitution to fund their drug/alcohol addiction behaviour [6, 10, 11], which often leads to entrapment of women who prostitute in this sex-for-drugs lifestyle [10] and others resort to drug/alcohol misuse to effectively sale their bodies without remorse [5, 11, 12]. Clearly, drug/alcohol misuse can increase prostitution with less safe sex [13], which in turn leads to increase in sexually transmitted infections (including HIV). Prostitution associated with drug/alcohol misuse is among the main factors in the spread of HIV in the United States. Similarly, in Mauritus 75% of the prostitutes reported injecting drugs and 23% said they never used nonsterile injecting equipment [14] which all pose a serious risk of HIV infection. Among drug misusing prostitutes in Mauritus who had been previously tested for HIV 13% were HIV positive, yet 68% of the prostitutes said they had not consistently used condoms during the previous three months [15]. HIV prevalence among female sex workers (prostitutes) varies widely but in some countries it is more than 20 times higher than the HIV prevalence of the general population [16].

A number of mathematical models have been developed to understand the role of social and behavioral processes in HIV transmission [17–19]. The later developed a cross impact model to explore intentional transmission of HIV by nondisclosure of status in various risky situations. Despite the fact that prostitution and HIV/AIDS are somehow linked, they have not been mathematically accounted for. Here following ideas generated by Bhunu and Mushayabasa [1], the authors offer an indepth analysis of prostitution and HIV/AIDS transmission from the mathematical persipective.

The rest of the manuscript is presented as follows. In Section 2, the model is presented and analysed. Numerical simulations are carried out in Section 3 and finally a discussion is presented.

## 2. Model Description

For this model the authors take a prostitute as anyone who buys sex to satisfy his/her own sexual desires or sells sex for material gain irregardless of that person's gender. The model subdivides the population based on prostitution and HIV/AIDS status. These are susceptibles who are not prostitutes *S*_*n*_(*t*), susceptible prostitutes *S*_*p*_(*t*), and HIV positive people not yet displaying AIDS symptoms and are not prostitutes *I*_*h*_(*t*), HIV positive prostitutes not yet displaying AIDS symptoms *I*_*h*_*p*__(*t*), HIV positive individuals who are not prostitutes and displaying AIDS symptoms *A*_*h*_(*t*), HIV positive prostitutes displaying AIDS symptoms *A*_*h*_*p*__(*t*), and finaly AIDS patients on treatment *A*_*h*_*t*__(*t*). The total population size is given by

(1) Nt=Sn(t)+Sp(t)+Ih(t)+Ihp(t)+Ah(t)+Ahp(t)+Aht(t).

It is assumed that susceptibles who are not prostitutes (*S*_*n*_(*t*)) are recruited into the population through birth at a constant rate Λ and that no person is born being a prostitute. Individuals in different human subgroups suffer from natural death at a constant rate *μ*, which is proportional to the number in each class. We assume that interaction is homogoneous. Although there are many causes of prostitution, here we dwell on peer pressure and poverty which are the main driving forces of prostitution among Zimbabweans [3]. The distinction between peer pressure and poverty as factors contributing to prostitution is not clear cut. Individuals who are not prostitutes turn into prostitution due to poverty related peer pressure at rate *β*_*p*_ to move into the corresponding classes of prostitutes. However, it is worth mentioning here that AIDS patients who have lost hope of living and are poverty stricken acquire prostitution habits at rate *σβ*_*p*_ with *σ* < [0,1) signifying the reduced chances an AIDS patient has of attracting clients due to the on and off sickness. Once they become prostitutes, they move into corresponding class of prostitutes displaying AIDS symptoms. We further assume that AIDS patients on treatment are nolonger prostitutes as they have been effectively counselled. Some prostitutes especially those driven by poverty leave the prostitution trade after getting meaningful employment and/or getting into financially stable relationship at rate *γ*_*p*_ to move into their corresponding nonprostituting classes. However, for those prostitutes in the AIDS stage of disease progression some are forced to leave prostitution due to sickness at rate *γ*_*s*_ as they are not likely to be gainfully employed or get a stable financially secure relationship while they are always on and off the sick bed.

Susceptible nonprostitutes and prostitutes acquire HIV following sexual contact with an infected individual at rates (1 − *ϵ*)*λ*(*t*) and (1 − *ϵ*)*κλ*_*h*_(*t*), respectively, to get into their corresponding HIV infected classes not yet showing AIDS symptoms (*I*_*h*_(*t*) and *I*_*h*_*p*__(*t*)). The role of condom use as a strategy to limit HIV infection is captured by *ϵ* ∈ (0,1) signifying that condom use offers some degree of protection against HIV infection and *κ* ≥ 1 signifies increased risk prostitutes have of contracting HIV infection as they often engage in less safe sex while they are under the influence alcohol and/or drugs [13]. The force of infection for HIV *λ*_*h*_(*t*) is given by

(2) λht=βhch(Ih(t)+Ihp(t)+θh(Ah(t)+Ahp(t))+ϕtAht(t))N(t),

where *β*_*h*_ is the probability of HIV transmission per sexual contact, *c*_*h*_ is the effective contact rate for HIV infection to occur, and *θ*_*h*_ > 1 models the fact that individuals in the AIDS stage and not on antiretroviral therapy are more infectious since the viral load is correlated with infectiousness [23]. It is assumed that individuals on antiretroviral therapy transmit infection at the smallest rate *ϕ*_*t*_ (with 0 < *ϕ*_*t*_ < 1) because of the fact that these individuals have very small viral load. It has been estimated by an analysis of longitudinal cohort data that antiretroviral therapy reduces per-partnership infectivity by as much as 60% (so that *ϕ*_*t*_ = 0.4) [22]. HIV infected nonprostitutes and prostitutes progress to the AIDS stage of disease progression at rates *ρ*_*n*_ and *ρ*_*p*_, respectively, with *ρ*_*n*_ ≤ *ρ*_*p*_ as prostitutes are more likely to experience multiple infections with other sexual transmitted infections, contributing to the more rapid deterioration of the immune system. We assume that antiretroviral therapy is given to AIDS individuals who are ill and have experienced AIDS-defining symptoms, or whose CD4+ T cell count is below 200/*μ*L, which is the recommended AIDS defining stage [23]. Thus, AIDS patients are assumed to get antiretroviral therapy at a constant rate *α*. Individuals in the AIDS stage and not yet on antiretroviral therapy experience AIDS related death at a rate *ν* > 0 and their corresponding parts on antiretroviral therapy eventually succumb to AIDS-induced mortality at a reduced rate *τν* with the parameter (0 < *τ* < 1). Unless stated otherwise, values for the parameters in the simulations are given in Table 1.

**Figure 1:**
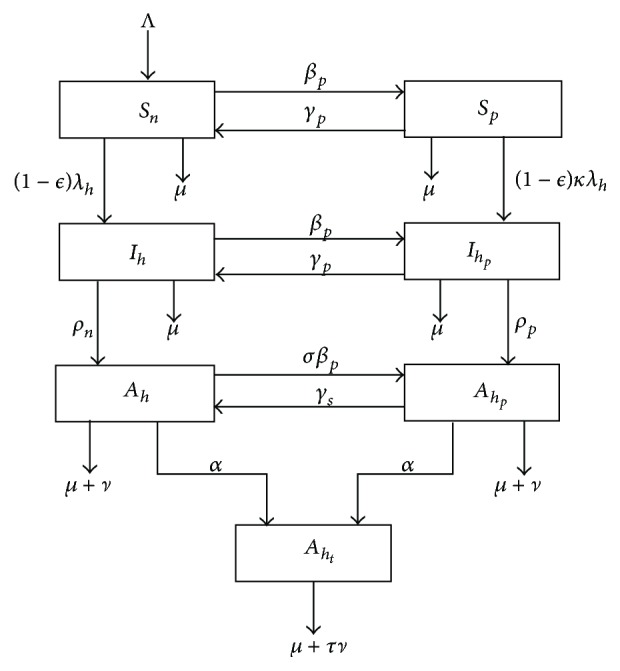
Structure of model.

The structure of the model is given in Figure 1.

Based on the aforementioned, the following system of differential equations describes the dynamics of prostitution and drug (alcohol) misuse:

(3) $$\begin{array}{c} S_{n}^{\text{'}}\left(t\right)=\Lambda -\left(1-\epsilon \right)\lambda_{h}S_{n}-\left(\beta_{p}+\mu \right)S_{n}+\gamma_{p}S_{p},\\ I_{h}^{\text{'}}\left(t\right)=\left(1-\epsilon \right)\lambda_{h}S_{n}-\beta_{p}I_{h}-\left(\mu +\rho_{n}\right)I_{h}+\gamma_{p}I_{h_{p}},\\ A_{h}^{\text{'}}\left(t\right)=\rho_{n}I_{h}-\sigma \beta_{p}A_{h}-\left(\mu +\nu +\alpha \right)A_{h}+\gamma_{s}A_{h_{p}},\\ S_{p}^{\text{'}}\left(t\right)=\beta_{p}S_{n}-\left(1-\epsilon \right)\kappa \lambda_{h}S_{p}-\left(\mu +\gamma_{p}\right)S_{p},\\ I_{h_{p}}^{\text{'}}\left(t\right)=\beta_{p}I_{h}+\left(1-\epsilon \right)\kappa \lambda_{h}S_{p}-\left(\mu +\rho_{p}+\gamma_{p}\right)I_{h_{p}},\\ A_{h_{p}}^{\text{'}}\left(t\right)=\sigma \beta_{p}A_{h}+\rho_{p}I_{h_{p}}-\left(\mu +\nu +\alpha +\gamma_{s}\right)A_{h_{p}},\\ A_{h_{t}}^{\text{'}}\left(t\right)=\alpha A_{h}+\alpha A_{h_{p}}-\left(\mu +\tau \nu \right)A_{h_{t}}. \end{array}$$

### 2.1. Basic Properties of the Model

In this section, we study some the basic results of solutions of model system (3) which are essential in the proofs of stability and persistence results.

**Lemma 1 .**

The nonnegative orthant *ℝ*_+_^7^ is positively invariant for model system (3).

**Proof**

Model system (3) can be written as *X*′ = *AX* + *B* with

(4) $$\begin{array}{c} A=\left[\begin{array}{ccccccc} -A_{1} & 0 & 0 & \gamma_{p} & 0 & 0 & 0\\ \left(1-\epsilon \right)\lambda_{h} & -A_{2} & 0 & 0 & \gamma_{p} & 0 & 0\\ 0 & \rho_{n} & -A_{3} & 0 & 0 & \gamma_{s} & 0\\ \beta_{p} & 0 & 0 & -A_{4} & 0 & 0 & 0\\ 0 & \beta_{p} & 0 & \left(1-\epsilon \right)\kappa \lambda_{h} & -A_{5} & 0 & 0\\ 0 & 0 & \sigma \beta_{p} & 0 & \rho_{p} & -A_{6} & 0\\ 0 & 0 & \alpha & 0 & 0 & \alpha & -A_{7} \end{array}\right],\;\;B=\left[\begin{array}{c} \Lambda \\ 0\\ 0\\ 0\\ 0\\ 0\\ 0 \end{array}\right]\\ A_{1}=\beta_{p}+\left(1-\epsilon \right)\lambda_{h}+\mu ,\;\;A_{2}=\beta_{p}+\mu +\rho_{n},\;\;A_{3}=\sigma \beta_{p}+\mu +\nu +\alpha ,\\ A_{4}=\left(1-\epsilon \right)\kappa \lambda_{h}+\mu +\gamma_{p},\;\;A_{5}=\mu +\rho_{p}+\gamma_{p},\;\;A_{6}=\mu +\nu +\alpha +\gamma_{s},\;\;A_{7}=\mu +\tau \nu . \end{array}$$

Note that *B* ≥ 0 and *A* is a Metzler matrix (*A* matrix is whose off-diagonal entries are nonnegative [24]) which implies that system (3) is positively invariant in *ℝ*_+_^7^.

**Lemma 2 .**

Each nonnegative solution is bounded in *L* ^1^-norm by max⁡{*N*(0), Λ/*μ*}.

**Proof**

The *L* ^1^-norm of each nonnegative solution *N* and it satisfies the inequality  *N*′ ≤ Λ − *μN*. Solutions to the equation *M*′ = Λ − *μM* are monotone increasing and bounded by Λ/*μ* if *M*(0) < Λ/*μ*. They are monotone decreasing and bounded above if *M*(0) ≥ Λ/*μ*. Since *N*′ ≥ *M*′, the claim follows.

**Corollary 3 .**

The region *Ω* = {(*S*_*n*_, *I*_*h*_, *A*_*h*_, *S*_*n*_, *I*_*h*_*p*__, *A*_*h*_*p*__, *A*_*h*_*t*__) ∈ *ℝ*_+_^7^ : *N* ≥ max⁡{*N*(0), Λ/*μ*}} is invariant and attracting for system (3).

Thus, the model is mathematically and epidemiologically well-posed and it is sufficient to consider the dynamics of the flow generated by the system (3) in *Ω*.

**Theorem 4 .**

For every nonzero, nonnegative initial value, solutions of model system (3) exist for all times.

**Proof**

Local existence of solutions follows from standard arguments, since the right hand side of system (3) is locally Liptschitz continuous. Global existence follows from* a priori* bounds.

### 2.2. Disease-Free Equilibrium and Stability Analysis

The disease-free equilibrium point of model system (3) is given by

(5) E0=Sn0,Ih0,Ah0,Sp0,Ihp0,Ahp0,Aht0=Λμ+γpμβp+μ+γp,0,0,Λβpμβp+μ+γp,0,0,0.

Following van den Driessche and Watmough [25] we have the reproduction number of the model as

(6) RHP=βhchκ1−ϵαϕt+θhm5ρpm1γs+m2+σβp2+m4ρnγph1m5m6m2+γsm1μ+ρp+μ+ρnγp +βhchh2(1−ϵ)(αϕt+θhm5)(βpρp(γs+m2)+(σβp+m4)ρnm3)h1m5m6(m2+γs)(m1(μ+ρp)+(μ+ρn)γp) +βhchh2(1−ϵ)(βp+m3)h1(m1(μ+ρp)+(μ+ρn)γp),

with *m*_1_ = *β*_*p*_ + *μ* + *ρ*_*n*_, *m*_2_ = *σβ*_*p*_ + *μ* + *ν* + *α*, *m*_3_ = *μ* + *ρ*_*p*_ + *γ*_*p*_, *m*_4_ = *μ* + *ν* + *α* + *γ*_*s*_, *m*_5_ = *μ* + *τν*, *m*_6_ = *μ* + *ν* + *α*, *h*_1_ = *β*_*p*_ + *μ* + *γ*_*p*_, and *h*_2_ = *h*_1_ − *β*_*p*_ throughout the paper. Here *ℛ*_*H*_*P*__ defines the number of secondary HIV cases generated by one HIV infected individual in community where prostitution is rife and condom use coupled with antiretroviral therapy is the intervention strategies available.  Theorem 5 follows from van den Driessche and Watmough [25] Theorem 2.

**Theorem 5 .**

The disease-free equilibrium *ℰ*_0_ is locally asymptotically stable for *ℛ*_*H*_*P*__ < 1 and unstable otherwise.

Using a theorem from Castillo-Chavez et al. [26], we show global stability when the reproduction number is less than unity.

**Theorem 6 .**

The disease-free equilibrium of system (3) is globally asymptotically stable provided that *ℛ*_*H*_*P*__ < 1.

**Proof**

Following Castillo-Chavez et al. [26], we write system (3) in the form:

(7) $$\begin{array}{c} X^{\text{'}}\left(t\right)=F\left(X,Y\right),\\ Y^{\text{'}}\left(t\right)=G\left(X,Y\right),\;G\left(X,0\right)=0, \end{array}$$

where *X* = (*S*_*n*_, *S*_*p*_) and *Y* = (*I*_*h*_, *A*_*h*_, *I*_*h*_*p*__, *A*_*h*_*p*__, *A*_*h*_*t*__). Here, *X* ∈ *ℝ*_+_^2^ denotes (its components) the number of uninfected individuals and *Y* ∈ *ℝ*_+_^5^ denotes (its components) the number of infected individuals. The disease-free equilibrium is now denoted by *ℰ*_0_ = (**X**_0_, 0), where **X**_0_ = (Λ*h*_2_/*μh*_1_, Λ*β*_*p*_/*μh*_1_). Here, we have to prove that the two conditions (*H*1) and (*H*2) are met.

(8) H1  For  X't=FX,0,            X0  is  globally  asymptotically  stableH2  GX,Y=UY−G^X,Y, G^X,Y≥0,                for  X,Y∈Ω.

Consider $F(X,0)=\left[\begin{array}{c} \Lambda -(\beta_{p}+\mu )S_{n}+\gamma_{p}S_{p}\\ \beta_{p}S_{n}-(\mu +\gamma_{p})S_{p} \end{array}\right]$,
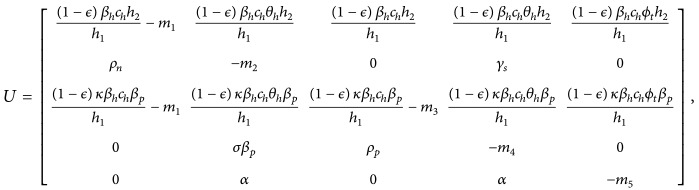
(9)

(10) $$\begin{array}{c} \hat{G}\left(X,Y\right)=\left[\begin{array}{c} \hat{G_{1}}\left(X,Y\right)\\ \hat{G_{2}}\left(X,Y\right)\\ \hat{G_{3}}\left(X,Y\right)\\ \hat{G_{4}}\left(X,Y\right)\\ \hat{G_{5}}\left(X,Y\right) \end{array}\right]=\left[\begin{array}{c} \left(1-\epsilon \right)\beta_{h}c_{h}\left(I_{h}+I_{h_{p}}+\theta_{h}\left(A_{h}+A_{h_{p}}\right)\phi_{t}A_{h_{t}}\right)\left(\frac{h_{2}}{h_{1}}-\frac{S_{n}}{N}\right)\\ 0\\ \left(1-\epsilon \right)\kappa \beta_{h}c_{h}\left(I_{h}+I_{h_{p}}+\theta_{h}\left(A_{h}+A_{h_{p}}\right)\phi_{t}A_{h_{t}}\right)\left(\frac{\beta_{p}}{h_{1}}-\frac{S_{p}}{N}\right)\\ 0\\ 0 \end{array}\right]. \end{array}$$

Clearly, $\hat{G_{2}}(X,Y)=\hat{G_{4}}(X,Y)=\hat{G_{5}}(X,Y)=0$ and so we have to show that $\hat{G_{1}}(X,Y)$ and $\hat{G_{3}}(X,Y)$ are both positive. We prove by contraction. Assume that statements in (11) are true,

(11) $$\begin{array}{c} \left(\text{i}\right)\;\;\frac{h_{2}}{h_{1}}<\frac{S_{n}}{N},\;\;\left(\text{ii}\right)\;\;\frac{\beta_{p}}{h_{1}}<\frac{S_{p}}{N}. \end{array}$$

From statements in (11) we have that

(12) h2+βph1 <Sn+SpN⟹N<Sn+Sd, since  h2+βph1=1,

and a contradiction as statement (12) is not true. Thus,

(13) $$\begin{array}{c} N\geq S_{n}+S_{p}⟹\left(\text{i}\right)\;\;\frac{h_{2}}{h_{1}}\geq \frac{S_{n}}{N},\;\;\left(\text{ii}\right)\;\;\frac{\beta_{p}}{h_{1}}\geq \frac{S_{p}}{N}. \end{array}$$

Thus, $\hat{G}(X,Y)\geq 0$. Therefore the disease-free equilibrium *ℰ*_0_ is globally asymptotically stable.

#### 2.2.1. Analysis of the Reproduction Number, *ℛ*_*H*_*P*__

The utility of the basic reproductive number has been questioned, but we use it here as a threshold with the understanding that there may be further complexities in its application [27]. Here we look into the following scenarios.

*Case  1i (every person is not prostitute).* In this case we have *β*_*p*_ = *γ*_*p*_ = *γ*_*s*_ = 0, *ρ*_*p*_ = *ρ*_*n*_, and *κ* = 1 and *ℛ*_*H*_*P*__ becomes

(14) RH=lim⁡(βp,γp,γs,κ,ρp)→(0,0,0,1,ρn)RHP=βhch(1−ϵ)μ+ρn1+ρn(αϕt+θhm5)(μ+1)μm5m6.

To check the impact of antiretroviral therapy on a prostitution-free environment we take the partial derivative of *ℛ*_*H*_ with respect to *α* to obtain

(15) ∂RH∂α=βhchρn1−ϵμ+1αϕt+θhμ+τν−μ+ν+αμm5m62(μ+ρn).

Equation (15) is negative only when 0 ≤ ((*θ*_*h*_ − 1)*μ* + (*τθ*_*h*_ − 1)*ν*)/(1 − *ϕ*_*t*_) < *α*. Thus, as long as 0 ≤ ((*θ*_*h*_ − 1)*μ* + (*τθ*_*h*_ − 1)*ν*)/(1 − *ϕ*_*t*_) < *α*, then antretroviral therapy may be able to keep HIV infections in check in a prostitution-free environment where condom use is in place. However, for 0 ≤ *α* < ((*θ*_*h*_ − 1)*μ* + (*τθ*_*h*_ − 1)*ν*)/(1 − *ϕ*_*t*_), the story is different as (15) will be positive, suggesting that the levels of antiretroviral therapy are not high enough to make a significant contribution to the to the reduction of HIV/AIDS cases.

*Case  1ii (effects of quitting prostitution).* Letting *γ*_*p*_ = *γ*_*s*_, then

(16) $$\begin{array}{c} R_{Q}=\underset{\gamma_{p}\to \infty }{\lim}R_{H_{P}}=\frac{\beta_{h}c_{h}\left(1-\epsilon \right)}{\mu +\rho_{n}}\left(\frac{\rho_{n}\alpha \phi_{t}}{m_{5}m_{6}}+\frac{\rho_{n}\theta_{h}}{m_{6}}+1\right),\\ \underset{\alpha \to \infty }{\lim}R_{Q}=\frac{\beta_{h}c_{h}\left(1-\epsilon \right)}{\mu +\rho_{n}}\left(\frac{\rho_{n}\theta_{h}}{m_{6}}+1\right). \end{array}$$

Clearly, *ℛ*_*Q*_ is decreasing function of *α* bounded above by (*β*_*h*_ *c*_*h*_(1 − *ϵ*)/(*μ* + *ρ*_*n*_))(*ρ*_*n*_ *θ*_*h*_/*m*_6_ + 1). This result suggests that antiretroviral therapy is more effective in communities with less levels of prostitution as there will be less chances of getting infected with different strains of HIV. Thus, prostitution should be targetted as a way of controlling the spread of multiple strains of HIV. Multiple strains of HIV may be difficult to keep in check using the commonly used antiretroviral therapy currently available.

*Case  2 (effects of condom use).* Clearly lim⁡_*ϵ*→1_ *ℛ*_*H*_*P*__ = 0 suggests even in the presence of prostitution increase in the consistant and correct use of condoms will be able to keep HIV infections under control. This result suggests that safe sex (condom use) should always be encouraged especially in areas where prostitution is endemic.

*Case  3 (every person becomes a prostitute).* This scenario happens when (*S*_*n*_^0^, *S*_*p*_^0^) → (0, Λ/*μ*) and (*β*_*p*_, *γ*_*p*_, *γ*_*s*_, *ρ*_*n*_)→(*∞*, 0,0, *ρ*_*p*_) so that *ℛ*_*H*_*P*__ becomes *ℛ*_*P*_*H*__ given by

(17) $$\begin{array}{c} R_{P_{H}}=\underset{\left(\beta_{p},\gamma_{p},\gamma_{s},\rho_{n})\to (\infty ,0,0,\rho_{p}\right)}{\lim}R_{H_{P}}=0. \end{array}$$

The fact that *ℛ*_*H*_*P*__ = 0 suggests if all people become prostitutes, then in the long term the entire population will be HIV infected (therefore no new HIV infections) as prostitutes often operate under the influence of drugs and/or alcohol resulting in inconstistancy in the use of condoms. This argument suggests that controlling prostitution may assist in keeping HIV infection in check. Furthermore, lim⁡_*κ*→*∞*_ *ℛ*_*H*_*P*__ = *∞* suggests that increase in the levels prostitution enhances the risk of acquiring HIV.

### 2.3. Endemic Equilibria and Stability Analysis

Theoretically, the following HIV/AIDS endemic equilibria exist: (i) the prostitution free-endemic equilibrium, (ii) the prostitution only endemic equilibrium, and (iii) equilibria where both prostitutes and nonprostitutes coexist. However, the first two are trivial cases and therefore are not going to be discussed here, so only the endemic equilibrium where HIV is in a population with both prostitutes and nonprostitutes is considered. This equilibrium point in terms of the force of infection *λ*_*h*_ is given by

(18) $$\begin{array}{c} E^{*}=\left(S_{n}^{*},I_{h}^{*},A_{h}^{*},S_{p}^{*},I_{h_{p}}^{*},A_{h_{p}}^{*},A_{h_{t}}^{*}\right) \end{array}$$

with the explicit expressions for *S*_n_^*^, *I*_h_^*^, *A*_h_^*^, *S*_p_^*^, *I*_*h*_p__^*^, *A*_*h*_p__^*^, and *A*_*h*_t__^*^ being cumbersome to be written explicitly. The permanence of the disease destabilizes the disease-free equilibrium *ℰ*_0_ since *ℛ*_*H*_*P*__ > 1, and the coexistence of the prostitutes and nonprostitutes endemic equilibrium *ℰ* ^*^ exists.

**Lemma 7 .**

System (3) is uniformly persistent on *Ω*.

**Proof**

Uniform persistence system of (3) implies there exists a constant *ζ* > 0 such that any solution of (3) which starts in $\overset{\circ }{\Omega }$ (the interior of *Ω*) remains in *Ω*. Also, $\left(S_{n}^{0},I_{h}^{0},A_{h}^{0},S_{p}^{0},I_{h_{p}},A_{h_{p}}^{0},A_{h_{t}}^{0}\right)\in \overset{\circ }{\Omega }$, and

(19) $$\begin{array}{c} \zeta \leq \underset{t\to \infty }{\mathrm{liminf}}S_{n}\left(t\right),\;\;\zeta \leq \underset{t\to \infty }{\mathrm{liminf}}I_{h}\left(t\right),\\ \zeta \leq \underset{t\to \infty }{\mathrm{liminf}}A_{h}\left(t\right),\;\;\zeta \leq \underset{t\to \infty }{\mathrm{liminf}}S_{p}\left(t\right),\\ \zeta \leq \underset{t\to \infty }{\mathrm{liminf}}I_{h_{p}}\left(t\right),\;\;\zeta \leq \underset{t\to \infty }{\mathrm{liminf}}A_{h_{p}}\left(t\right),\\ \zeta \leq \underset{t\to \infty }{\mathrm{liminf}}A_{h_{t}}\left(t\right). \end{array}$$

Define the following Korobeinikov-Maini [28] type Lyapunov function

(20) V(Sn,Ih,Ah,Sp,Ihp,Ahp,Aht) =(Sn−Sn∗ln⁡Sn)+(Ih−Ih∗ln⁡Ih)+(Ah−Ah∗ln⁡Ah)  +(Sp−Sp∗ln⁡Sp)+(Ihp−Ihp∗ln⁡Ihp)  +Ahp−Ahp∗ln⁡Ahp+Aht−Aht∗ln⁡Aht,

which is continuous for all (*S*_*n*_, *I*_*h*_, *A*_*h*_, *S*_*p*_, *I*_*h*_*p*__, *A*_*h*_*p*__, *A*_*h*_*t*__) > 0 and satisfies (cf. [29])

(21) $$\begin{array}{c} \frac{\partial V}{\partial S_{n}}=\left(1-\frac{S_{n}^{*}}{S_{n}}\right),\ldots ,\frac{\partial V}{\partial A_{h_{t}}}=\left(1-\frac{A_{h_{t}}^{*}}{A_{h_{t}}}\right). \end{array}$$

Consequently, the endemic equilibrium *ℰ* ^*^ is the only extremum and the global minimum of the function *V* ∈ *ℝ*_+_^7^. Also, *V*(*S*_*n*_, *I*_*h*_, *A*_*h*_, *S*_*p*_, *I*_*h*_*p*__, *A*_*h*_*p*__, *A*_*h*_*t*__) > 0 and *V*′(*S*_*n*_, *I*_*h*_, *A*_*h*_, *S*_*p*_, *I*_*h*_*p*__, *A*_*h*_*p*__, *A*_*h*_*t*__) = 0 only at *ℰ* ^*^. Thus, *V*(*S*_*n*_, *I*_*h*_, *A*_*h*_, *S*_*p*_, *I*_*h*_*p*__, *A*_*h*_*p*__, *A*_*h*_*t*__) is a Lyapunov function. At equilibrium, Λ = (*λ*_h_^*^(1 − *ϵ*) + *β*_*p*_ + *μ*)*S*_n_^*^ − *γ*_*p*_ *S*_p_^*^, substituting this into the time derivative of *V* along the solution path of model system (3), we have

(22) V'=(Sn−Sn∗)Sn'Sn+(Ih−Ih∗)Ih'Ih+(Ah−Ah∗)Ah'Ah+(Sp−Sp∗)Sp'Sp+Ihp−Ihp∗Ihp'Ihp+(Ahp−Ahp∗)Ahp'Ahp+(Aht−Aht∗)Aht'Aht≤−μSn−Sn∗2Sn+gSn,Ih,Ah,Sp,Ihp,Ahp,Aht,

where *g* can be shown to be nonpositive using Barbalat Lemma [30]. Hence, *V*′(*S*_*n*_, *I*_*h*_, *A*_*h*_, *S*_*p*_, *I*_*h*_*p*__, *A*_*h*_*p*__, *A*_*h*_*t*__) ≤ 0 with equality only at *ℰ* ^*^. The only invariant set in $\overset{\circ }{\Omega }$ is the endemic equilibrium *ℰ* ^*^. Thus, all solutions of (3) which intersect $\overset{\circ }{\Omega }$ converge to the invariant (singleton) {*ℰ* ^*^}. Therefore, from Lyapunov-Lasalle invariance principle, system (3) is uniformly persistent.

## 3. Numerical Simulations

In this section, we make use of Matlab to analyse model system (3) using model parameters in Table 1 and the following initial conditions: *S*_*n*_(0) = 3 · 10^6^, *S*_*p*_(0) = 5 · 10^5^, *I*_*h*_(0) = 3 · 10^5^, *I*_*h*_*p*__(0) = 2 · 10^5^, *A*_*h*_(0) = 10^3^, *A*_*h*_*p*__(0) = 10^3^, and *A*_*h*_*t*__(0) = 0.

**Figure 2:**
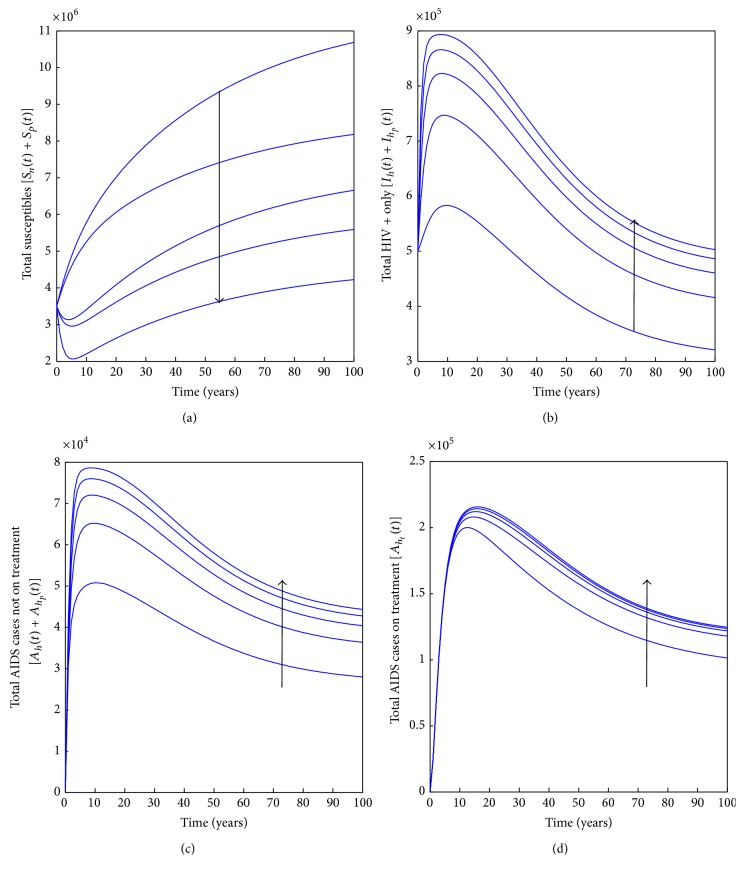
Simulations of model system (3) showing the effects of prostitution on the HIV and AIDS pandemic as the percentage of prostitutes is varied from 0% to 100% with a step size of 25%. The direction of the arrow shows the direction of increase. Parameter values used are in Table 1.

In Figure 2, the possible effects prostitution has on HIV levels are noted by varying the percentages of prostitutes in the community in the presence of antiretroviral therapy and condom use. It is noted that an increase in the percentage of prostitutes results in an increase in the number of HIV infected people as prostitutes mostly operate under the influence of drugs and therefore have difficulties to properly use condoms. This in turn results in the number of AIDS cases in the population. This suggests increase in employment creation coupled with counselling will be able to keep the prostitution in check as most people resort to prostitution due to poverty. This result somehow points to poverty as driving prostitution which in turn drives HIV in poor settings. Thus, poverty eradication reduces prostitution which in turn results in a reduction in the number of HIV infections in the population.

**Figure 3:**
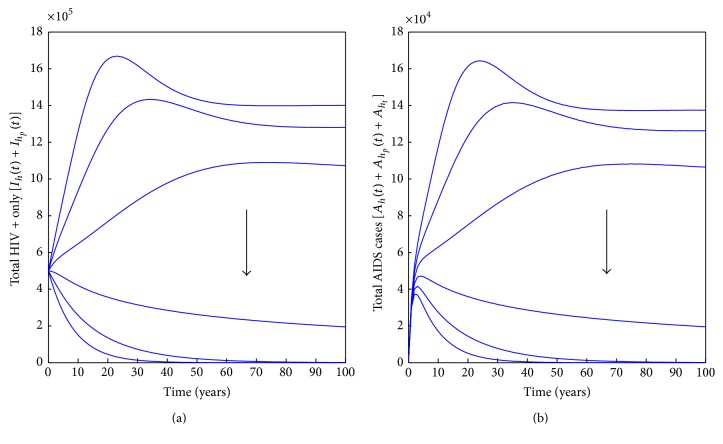
Simulations of model system (3) for varying rates of condom use. The direction of the arrow shows the direction of increase in the rate of proper condom use starting at *ϵ* = 0 and increasing with step size of 0.2. Parameter values used are in Table 1.

In Figure 3, it is shown that proper use of condoms will be able to keep HIV infections in check, even when prostitution exists in the population. This result suggests that encouraging the proper use of condoms should be encouraged especially in communities where prostitution is rife. It is important to note that proper use of condoms may be difficult especially among prostitutes as they often engage in sex while they are under the influence of drugs/alcohol.

## 4. Discussion

Prostitution is an important driver of HIV/AIDS in most resource constrained settings. A mathematical model for the effect of prostitution on the transmission dynamics of HIV/AIDS is presented as a system of differential equations. The reproduction number of the model is computed and analysed. The disease-free equilibrium point of the model is shown to be globally asymptotically stable when the corresponding reproduction number is less than unity. Results from the analysis of the reproduction number suggest the following. (i) Increase in the levels of prostitution in a community enhances the chances of acquiring HIV if not infected and acquiring another HIV strain if already infected. (ii) Antiretroviral therapy is more effective in communities with less prostitution as less prostitution corresponds to reduced chances of one getting infected with different strains of HIV. Infection with different strains of HIV makes the HIV infected person worse as some strains may not respond to the antiretroviral therapy regimen available especially in resource-constrained settings. (iii) Increase in the rates of proper condom use may be able to keep HIV infections even in communities where prostitution is rife.

Numerical simulations are performed to illustrate various dynamical regimes. Graphical representations clearly show that the number of HIV infected people and those displaying AIDS symptoms is higher in communities with more prostitutes than in communities with less prostitutes. This suggests that prostitution promotes HIV transmission, a result in total agreement with the analytical results. Furthermore, graphical representations show increased reduction in levels of HIV infection when proper condom use is in place. Thus, in the absence of monitored proper condom use which is the case in the world as sexual intercourse is a private thing, there is a strong need to control prostitution. Even in the presence antiretroviral therapy, HIV/AIDS control is more effective in less-prostituting communities than in the prostituting communities. Thus, prostitution may reduce the effectiveness of antiretroviral therapy by way of acquiring multiple strains of HIV as prostitutes often operate under the influence of alcohol/drugs and hence fail to engage in safe sex. Hence, prostitution negatively affects HIV control and as long as HIV control is taken as a biomedical intervention only; controlling HIV through antiretroviral therapy alone may not be successful in populations where prostitution is common. To put prostitution in check, there may be target poverty as reducing it will mean less people moving into prostitution as most are driven into it by poverty. It is worth mentioning here that the study presented here is not exhaustive, and it can be extended to incorporate alcohol/drug misuse which has always been associated with both prostitution and HIV/AIDS. The model may be further modified to assess the impact of poverty alleviation in HIV control.

## Figures and Tables

**Table 1:** Model parameters and their interpretations.

Definition	Symbol	Units	Point estimate	Range	Source
Recruitment rate	Λ	People/year	3.48 · 10^5^	—	CSOZ

Natural mortality rate	*μ*	1/year	0.02	0.015–0.02	CSOZ

AIDS induced death rate	*ν*	1/year	0.34	0.33–0.4	a^*^

Rate of becoming a prostitute	*β*_*p*_	1/year	0.025	0-1	Assumed

Product of effective HIV contact rate and probability of HIV transmission per contact	*β*_*h*_ *c*_*h*_	1/year	0.025	0.011–0.95	b^*^

Rate of quitting prostitution due to poverty eradication and sickness	*γ*_*p*_, *γ*_*s*_	1/year	0.5	0.1–1	d^*^

Natural rate of progression to AIDS	*ρ*_*p*_, *ρ*_*n*_	1/year	0.1	0.05–0.25	a^*^

Enhancement factor	*κ*	—	1.25	(≥1)	Assumed

Enhancement factor	*θ*_*h*_	—	1.02	(≥1)	a^*^

Reduction factor	*ϕ*_*t*_	—	0.4	0–0.75	c^*^

Reduction factor	*τ*	—	0.5	0-1	Assumed

Here, CSOZ stands for Central Statistics Office of Zimbabwe; a^*^ stands for parameter values adapted from Bhunu et al., 2009 [20]; b^*^ stands for parameter values adapted from Hyman et al., 1999 [21]; c^*^ stands for parameter values adapted from Porco et al., 2004 [22]; d^*^ parameter values adapted from Bhunu and Mushayabasa 2012 [1].

## References

[1] Bhunu C. P., Mushayabasa S. (2012). Prostitution and drug (alcohol) misuse: the menacing combination. *Journal of Biological Systems*.

[2] Thappa D. M., Singh N., Kaimal S. (2007). Prostitution in India and its role in the spread of HIV infection. *Indian Journal of Sexually Transmitted Diseases*.

[3] Makumbirofa C. Gambia Affairs: Gambia: Massive Pros titution By Zimbabwean Women in South Africa. http://gambiaaffairs.blogspot.com/2011/09/gambia-affairsgambiamassive.html.

[4] Ringdals N. J. (2004). *Love for Sale: A World History of Prostitution*.

[5] Silbert M. H., Pines A. M., Lynch T. (1982). Substance abuse and prostitution. *Journal of Psychoactive Drugs*.

[6] Kuhns J. B., Heide K. M., Silverman I. (1992). Substance use/misuse among female prostitutes and female arrestees. *The International Journal of the Addictions*.

[7] El-Bassel N., Schilling R. F., Irwin K. L. (1997). Sex trading and psychological distress among women recruited from the streets of Harlem. *The American Journal of Public Health*.

[8] Nadon S. M., Koverola C., Schludermann E. H. (1998). Antecedents to prostitution: childhood victimization. *Journal of Interpersonal Violence*.

[9] Potterat J. J., Rothenberg R. B., Muth S. Q., Darrow W. W., Phillips-Plummer L. (1998). Pathways to prostitution: the chronology of sexual and drug abuse milestones. *The Journal of Sex Research*.

[10] Gossop M., Powis B., Griffiths P., Strang J. (1994). Sexual behaviour and its relationship to drug-taking among prostitutes in South London. *Addiction*.

[11] Weeks M. R., Grier M., Romero-Daza N., Puglisi-Vasquez M. J., Singer M., Stevens S. J., Tortu S., Coyle S. L. (1998). Streets, drugs, and the economy of sex in the age of AIDS. *Women, Drug Use and HIV Infection*.

[12] Young A. M., Boyd C., Hubbell A. (2000). Prostitution, drug use and copi ng with psychological distress. *Journal of Drug Issues*.

[13] Morrison C. L., McGee A., Ruben S. M. (1995). Alcohol and drug misuse in prostitutes. *Addiction*.

[14] Sulliman F. T., Sag A. (2004). Mauritius epidemiology network for drug use report.

[15] Sulliman F. T., Ameerberg S. A. G., Dhanoo M. I. (2004). *Report of the Rapid Situation Assessment and Responses on Drug Use in Mauritius and Rodrigues*.

[16] UNAIDS/WHO

[17] Kimbir A. R., Oduwole H. K. (2008). A mathematical model of HIV/AIDS transmission dynamics considering counselling and antiretroviral therapy. *Journal of Modern Mathematics and Statistics*.

[18] Ajay M., Brendan O., David E. B. Needle sharing and HIV transmission: a model with markets and purposive behavior. http://ideas.repec.org/p/nbr/nberwo/14823.html.

[19] Pedamallu C. S., Ozdamar L., Kropat E., Weber G.-W. (2012). A system dynamics model for intentional transmission of HIV/AIDS using cross impact analysis. *CEJOR: Central European Journal of Operations Research*.

[20] Bhunu C. P., Garira W., Magombedze G. (2009). Mathematical analysis of a two strain HIV/AIDS model with antiretroviral treatment. *Acta Biotheoretica*.

[21] Hyman J. M., Li J., Ann Stanley E. (1999). The differential infectivity and staged progression models for the transmission of HIV. *Mathematical Biosciences*.

[22] Porco T. C., Martin J. N., Page-Shafer K. A. (2004). Decline in HIV infectivity following the introduction of highly active antiretroviral therapy. *AIDS*.

[23] WHO (2005). *Guidelines for HIV Diagnosis and Monitoring of Anti Retroviral Therapy*.

[24] Berman A., Plemmons R. J. (1994). *Nonnegative Matrices in the Mathematical Sciences*.

[25] van den Driessche P., Watmough J. (2002). Reproduction numbers and sub-threshold endemic equilibria for compartmental models of disease transmission. *Mathematical Biosciences*.

[26] Castillo-Chavez C., Feng Z., Huang W., Castillo-Chavez C., Blower S., van den Driessche P., Kirschner D., Yakubu A.-A. (2002). On the computation of R0 and its role on global stability. *Mathematical Approaches for Emerging and Reemerging Infectious Diseases: An Introduction*.

[27] Li J., Blakeley D., Smith R. J. (2011). The failure of *R*
_0_. *Computational and Mathematical Methods in Medicine*.

[28] Korobeinikov A., Maini P. K. (2004). A Lyapunov function and global properties for SIR and SEIR epidemiological models with nonlinear incidence. *Mathematical Biosciences and Engineering*.

[29] Korobeinikov A., Wake G. C. (2002). Lyapunov functions and global stability for SIR, SIRS, and SIS epidemiological models. *Applied Mathematics Letters*.

[30] Barbalat I. (1959). Systeme d'equations différentielles d'oscillation nonlinéaires. *Revue Roumaine de Mathématique Pures et Appliquées*.

